# Melanoma-specific bcl-2 promotes a protumoral M2-like phenotype by tumor-associated macrophages

**DOI:** 10.1136/jitc-2019-000489

**Published:** 2020-04-07

**Authors:** Marta Di Martile, Valentina Farini, Francesca Maria Consonni, Daniela Trisciuoglio, Marianna Desideri, Elisabetta Valentini, Simona D'Aguanno, Maria Grazia Tupone, Simonetta Buglioni, Cristiana Ercolani, Enzo Gallo, Bruno Amadio, Irene Terrenato, Maria Laura Foddai, Antonio Sica, Donatella Del Bufalo

**Affiliations:** 1 Preclinical Models and New Therapeutic Agents Unit, IRCCS Regina Elena National Cancer Institute, Rome, Italy; 2 Molecular Immunology Lab, Humanitas Clinical and Research Center, Milan, Italy; 3 Institute of Molecular Biology and Pathology, National Research Council, Rome, Italy; 4 Department of Life, Health and Environmental Sciences, University of L'Aquila, L'Aquila, Italy; 5 Pathology Unit, IRCCS Regina Elena National Cancer Institute, Rome, Italy; 6 SAFU Unit, IRCCS Regina Elena National Cancer Institute, Rome, Italy; 7 Biostatistics and Bioinformatic Unit-Scientific Direction, IRCCS Regina Elena National Cancer Institute, Rome, Italy; 8 Immunohematology and Transfusional Medicine Unit, IRCCS Regina Elena National Cancer Institute, Rome, Italy; 9 Department of Pharmaceutical Sciences, Università del Piemonte Orientale “Amedeo Avogadro”, Novara, Italy

**Keywords:** melanoma, macrophages, tumor microenvironment

## Abstract

**Background:**

A bidirectional crosstalk between tumor cells and the surrounding microenvironment contributes to tumor progression and response to therapy. Our previous studies have demonstrated that bcl-2 affects melanoma progression and regulates the tumor microenvironment. The aim of this study was to evaluate whether bcl-2 expression in melanoma cells could influence tumor-promoting functions of tumor-associated macrophages, a major constituent of the tumor microenvironment that affects anticancer immunity favoring tumor progression.

**Methods:**

THP-1 monocytic cells, monocyte-derived macrophages and melanoma cells expressing different levels of bcl-2 protein were used. ELISA, qRT-PCR and Western blot analyses were used to evaluate macrophage polarization markers and protein expression levels. Chromatin immunoprecipitation assay was performed to evaluate transcription factor recruitment at specific promoters. Boyden chamber was used for migration experiments. Cytofluorimetric and immunohistochemical analyses were carried out to evaluate infiltrating macrophages and T cells in melanoma specimens from patients or mice.

**Results:**

Higher production of tumor-promoting and chemotactic factors, and M2-polarized activation was observed when macrophages were exposed to culture media from melanoma cells overexpressing bcl-2, while bcl-2 silencing in melanoma cells inhibited the M2 macrophage polarization. In agreement, the number of melanoma-infiltrating macrophages in vivo was increased, in parallel with a greater expression of bcl-2 in tumor cells. Tumor-derived interleukin-1β has been identified as the effector cytokine of bcl-2-dependent macrophage reprogramming, according to reduced tumor growth, decreased number of M2-polarized tumor-associated macrophages and increased number of infiltrating CD4^+^IFNγ^+^ and CD8^+^IFNγ^+^ effector T lymphocytes, which we observed in response to in vivo treatment with the IL-1 receptor antagonist kineret. Finally, in tumor specimens from patients with melanoma, high bcl-2 expression correlated with increased infiltration of M2-polarized CD163^+^ macrophages, hence supporting the clinical relevance of the crosstalk between tumor cells and microenvironment.

**Conclusions:**

Taken together, our results show that melanoma-specific bcl-2 controls an IL-1β-driven axis of macrophage diversion that establishes tumor microenvironmental conditions favoring melanoma development. Interfering with this pathway might provide novel therapeutic strategies.

## Background

Melanoma represents the most deadly form of skin cancer, and molecular mechanisms leading to its development and progression are the focus of intense investigation aimed at developing new treatment strategies. A number of driver mutations have been identified and the most common mutations affect the Ras/Raf/mitogen-activated protein kinase pathways. Further, the bcl-2 network is found dysregulated in melanoma, where high bcl-2 levels account for cancer stem cells mediated therapeutic resistance to apoptosis and poor prognosis.^1–3^

Using in vitro and in vivo preclinical models, we previously found that, in addition to its canonical antiapoptotic role, bcl-2 modulation in human melanoma cells regulates tumor progression-associated properties and tumor metastatization through a BH4 domain-dependent mechanism.^4–6^ Recently, we also described that bcl-2-mediated modulation of microRNA-211 regulates melanoma cells migration and the activity of microphthalmia-associated transcription factor.^7^

Tumor growth and metastases are determined by the complex crosstalk between tumor cells and the surrounding microenvironment, through secretion of tumorigenic mediators. In this scenario, it has been reported that bcl-2 plays a pivotal role in orchestrating the crosstalk between tumor and neovascular endothelial cells. In particular, we showed that bcl-2 overexpression in cancer cells cooperates with hypoxia to increase transcriptional activity of hypoxia inducible factor 1 and expression of vascular endothelial growth factor (VEGF).^5 8^

Tumor-associated macrophages (TAM) are pivotal in affecting the nature of the tumor microenvironment and can induce both positive and negative effects on tumor growth.^9^ In particular, TAM promote tumor progression^10^ supporting tumor cell migration/invasion,^11^ angiogenesis,^12^ immune suppression and drug resistance.^13^ TAM infiltration has been found to directly correlate with melanoma thickness^14^ and with increased angiogenesis and microvessel density, through modulation of tumor proinflammatory factors.^15^ Moreover, elevated number of TAM in the tumor microenvironment is often correlated with poor prognosis in melanoma.^16^ Thus, macrophages represent promising therapeutic targets and their depletion can be an effective therapeutic intervention in the management of melanoma.^15 16^

Melanoma cells and TAM interact with each other through cell contact-mediated signals or through the release of soluble factors: metastatic melanoma cells have been reported to produce cytotoxic substances against macrophage that help tumor cells to escape the host immunosurveillance system and to prevent distant metastasis,^14^ while macrophages have been found to transfer cytoplasmic molecules to melanoma cells or to fuse with them with a consequence of enhanced in vivo melanoma cell dissemination.^17 18^

In addition to its ability to increase melanoma, and more in general, tumor cell survival, bcl-2 also favors the survival of immune cells including macrophages:^19^ Triggering receptor expressed on myeloid cells 1 has been reported to prolong macrophage survival through bcl-2 regulation, while estrogen induced bcl-2 upregulation has been identified as a mechanism necessary for macrophages survival.^20^ In this context, targeting bcl-2 in order to interrupt the liaison between tumor cells and tumor microenvironment is emerging as a new interesting therapeutic strategy.^21^ However, no comprehensive information exists on the effects elicited by tumor-specific bcl-2 on TAM accumulation or, more in general, on tumor infiltrating cells. Understanding the mechanistic basis of this interaction would likely lead to generation of more effective therapies. Our data demonstrate, for the first time, a synergy among bcl-2 overexpressing melanoma cells and cellular components of tumor microenvironment.

## Materials and methods

### Cell cultures and treatments

Human melanoma M14, A375SM-SC1, lung cancer H1299 and monocytic THP-1 cell lines were cultured in RPMI 1640 medium (Euroclone, Milan, Italy), while the murine melanoma B16/F10 in DMEM medium (LONZA, Verviers, Belgium) supplemented with 10% inactivated fetal bovine serum (Hyclone, Thermoscientific, South Logan, Utah, USA), 1% L-glutamine and 100 µg/mL penicillin/streptomycin (Euroclone).

Human control and bcl-2 overexpressing stable cells were obtained from parental cells as previously reported.^7 22^ Murine control and bcl-2 overexpressing stable cells were obtained by transfecting B16/F10 melanoma cells with JetPrime (PolyPlus Transfection, Illkirch, France) and culturing them with G418 (800 µg/mL, Euroclone). For siRNA transfection, cells were seeded and, after 24 hours, transfected with 20 nM pooled siRNA oligonucleotides against bcl-2 (si-bcl-2) or control (si-control) sequences (siGENOME SMART pool, DharmaconRNA Technologies, Lafayette, Colorado, USA), by using JetPrime. Transient transfection for the expression of S32A/S36A mutated IκBα (IκBSR) protein was performed using JetPrime. Expression vectors encoding the murine bcl-2 protein and human IκBSR were kindly provided by Taglialatela G and Cippitelli M, respectively. For experiments with neutralizing antibodies, cells were treated with human anti-interleukin-1β (IL-1β, 0.2 µg/mL), interleukin-17 (IL-17, 0.5 µg/mL), or interleukin-8 (IL-8, 0.2 µg/mL) (R&D Systems, Minneapolis, Minnesota, USA), antibodies for 24 hours.

### Monocytes isolation and differentiation

THP-1 monocytes were differentiated in macrophages with 100 ng/mL phorbol-12-myristate-13-acetate (PMA, Sigma-Aldrich, San Louis, USA) for 24 hours. Human monocytes were isolated from healthy donor buffy coats by using Lympholite-H (Euroclone). Buffy coats were provided by the Immunohematology and Transfusional Medicine Unit of our Institute. Purified monocytes were incubated for 10 days in RPMI 1640 supplemented with 10% inactivated fetal bovine serum and 50 ng/mL macrophage colony-stimulating factor (Peprotech, London, UK) to obtain mature monocyte-derived macrophages (M-DM). M-DM were stimulated for 24 hours with serum free medium (M0 macrophages) or with culture medium (CM) derived from control, bcl-2 overexpressing or bcl-2 silenced melanoma cells. In all experiments, the CM used for stimulating M-DM was normalized to the number of adherent cells.

### Cell migration assay

1×10^5^ THP-1 cells were plated in the upper chamber of Transwell (Costar, New York, USA) containing 5 µm pore polycarbonate membrane. CM derived from control or bcl-2 overexpressing cells were added in the lower chamber. After 3 hours, cells remaining on the top side of the membrane were removed and migrating cells were fixed, stained (Differential Quick Stain Kit, Dade Behring, Marburg, Germany), photographed by using light microscopy, and quantified by counting the number of migrated cells in 10 images for each condition.

### Elisa and Western blot analyses

ELISA was used to evaluate interleukin-10 (IL-10), interleukin-12 (IL-12, R&D Systems), prostaglandin E2 (PGE2, Elabscience Biotechnology), IL-1β, IL-8 and IL-17 (Enzo Life Sciences, New York, USA) content in CM derived from melanoma cells or macrophages. Following manufacturer’s instructions, each sample was evaluated in duplicate and protein levels were normalized to the number of adherent cells.

Western blot analyses of total extracts were performed as previously described.^4^ Nuclear and cytoplasmic fractions were obtained by using the nuclear and cytoplasmic extraction kit (Thermo Scientific, Rockford, Illinois, USA) following the manufacturer’s instructions. Immunodetection was performed using antibodies directed to β-actin (Sigma-Aldrich), Cyclooxygenase 2 (COX-2, Cayman Chemical, Ann Arbor, Michigan, USA), histone H3 (Cell Signaling, Danvers, USA), human bcl-2 (100), p65, IKBα and murine bcl-2 (10C4) (Santa Cruz Biotechnology, Dallas, Texas, USA), HSP72/73 (Calbiochem, San Diego, California, USA), HSP90 (BD biosciences, San Diego, California, USA). Anti-rabbit or anti-mouse IgG-horseradish peroxidase-conjugated antibodies (Amersham Biosciences, Freiburg, Germany) were used as secondary antibody. Antibody binding was visualized by enhanced chemiluminescence method according to manufacturer’s specification and recorded on autoradiographic film (Amersham Biosciences). Densitometric evaluation was performed using Image J software and normalized with relative β-actin, HSP72/73, HSP90 or H3 expression.

### RNA extraction and qRT-PCR

Total RNA was extracted using a Qiagen RNeasy Mini kit (Qiagen, Hilden, Germany) and Reverse transcription was performed using RevertAid Reverse Transcriptase (Thermo Scientific) kit and Gene-Amp 9700 PCR system (Applied Biosystems, Foster City, California, USA). qRT-PCR was performed using 7900HT Fast Real Time PCR system (Applied Biosystems), using the SYBR green dye detection method. The mRNA levels were normalized using β-actin. Primers used to analyze each gene are listed in online supplementary table S1. The results were evaluated by the ΔΔCt method.

10.1136/jitc-2019-000489.supp1 Supplementary data

10.1136/jitc-2019-000489.supp2 Supplementary data

**Table 1:** Macrophage depletion reduces tumor appearance in bcl-2 derived allografts

	Days			
	8	11	14	18
Control+vehicle	7/8 (87.5%)	8/8 (100%)	8/8 (100%)	8/8 (100%)
Bcl-2+vehicle	8/8 (100%)	8/8 (100%)	8/8 (100%)	8/8 (100%)
Bcl-2+Clodronate	5/8 (62.5%)	6/8 (75%)	6/8 (75%)	7/8 (87.5%)

The number of mice with tumors/number of mice (% of tumor appeared) is indicated.

### Chromatin immunoprecipitation (ChIP) assay

2×10^6^ cells were plated onto 150 mm dishes. After 48 hours, chromatin was cross-linked with 1% formaldehyde and sonicated. Chromatin was immunoprecipitated overnight with anti-p65, anti-polimerase II (Pol II) (Santa Cruz Biotechnology) or anti-acetylated H3 (Millipore, Billerica, Massachusetts, USA) antibodies. Primers used for the ChIP were indicated in online supplementary table S2. Quantization of immunoprecipitated DNA was performed on the 7500 Real-Time PCR System (Applied Biosystems), using the SYBR green dye detection method. The results were evaluated by the ΔΔCt method. To identify the binding sites of p65 on the IL-1β, IL-8, COX-2 and monocyte-chemotactic protein 1 (MCP1/CCL2) promoters, we used MATCH_SEARCH, MatINSPECTOR and LASAGNA tools.

10.1136/jitc-2019-000489.supp3 Supplementary data

### *In vivo* experiments

5×10^6^ M14 control or bcl-2 overexpressing cells were subcutaneously injected into 6–8-week-old female immunodeficient athymic CD1 nude mice and euthanized 15 or 30 days after injection. 2×10^5^ B16/F10 control or bcl-2 overexpressing cells were injected subcutaneously in 6–8-week-old female C57/Bl6 mice and sacrificed 19 days after injection. For kineret (anakinra, Sobi, Stockholm, Sweden) treatment, 3 days after cell injection mice were treated i.p. with vehicle or with kineret 1 mg/kg daily for 10 days. For clodronate liposomes (Clophosome, FormuMax Scientific, Sunnyvale, California, USA) treatment, 3 days after cell injection mice were treated intravenously with vehicle or clodronate (200 ul) two times a week up to the day of the sacrifice. Mice were monitored for any signs of pain, and tumor growth was monitored using a caliper.

### Immunohistochemical (IHC) analysis

Immediately after mice sacrifice, tumors were fixed in 4% buffered formalin and paraffin embedded. Immunoreactions were revealed by Bond Polymer Refine Detection in an automated autostainer (BondTM Max, Leica Biosystems, Milan, Italy) using anti-F4/80 (SP115, ThermoFisher), -CD206 (Abcam, Cambridge, UK) or -bcl-2 (124, Dako, Milan, Italy) antibodies. For each tumor, three different 2 µm paraffin sections were analyzed and examined by light microscopy. The presence of F4/80 or CD206 positive cells was classiﬁed as peritumoral (PT) or intratumoral (IT) depending on their localization in the stroma surrounding the tumor islands or within the tumor-cell nests, respectively. IT-positive or PT-positive cells were counted in four high-power ﬁelds (HPF) per section and averages of positive cells/HPF in both compartments were calculated. Tumors were classiﬁed into four categories depending on the average number of cells/HPF (score 0: absence of positive cells; score 1: presence of <5 positive cells/HPF; score 2: presence of ≥5≤10 positive cells/HPF; score 3: presence of >10 positive cells/HPF). Evaluation of the IHC results was performed independently and in blinded manner by two investigators at 200× and 400× magnifications.

### Cytofluorimetric analysis of immune cell infiltrate

Tumors were minced with scissors and incubated in DMEM medium (Euroclone) containing Collagenase IV (0.5 mg/mL) and DNase I (20 µg/mL) (Sigma-Aldrich) for 20 min at 37°C. Cells were resuspended in Hank’s balanced salt solution (Lonza) supplemented with 0.5% BSA (Sigma-Aldrich) and stained with the combination of specific antibodies: CD45-PerCP (30-F11); CD11b-FITC, -PE-Cy7, -BV711 (M1/70); F4/80-PE (BM8); CD206-APC (C06C2); MHCII-BV480 (M5/114.15.2); CD3-PE, -BV650 (145–2 C11); CD4-PECy7 (GK1.5); CD8-APC, -PECy5 (53–6.7); IFNγ-FITC (XMG1.2); CD44-FITC (IM7), CD62L-APC, -BV570 (MEL-14) from BD Bioscience, eBioscience (Thermo Fisher Scientific) or BioLegend (San Diego, California, USA). All surface markers were stained for 20 min at 4°C; intracellular detection of IFNγ and CD206 was performed following fixation of cells with Foxp3/Transcription Factor Staining Buffer Set (eBioscience) according to the manufacturer’s instructions and incubating with specific antibodies for 30 min at 4°C. The expression levels of IFNγ were analyzed after stimulation for 3 hour with brefeldin A (1 µg/mL), ionomycin (1 µg/mL) and PMA (5 ng/mL) (Sigma-Aldrich). Cell viability was determined by LIVE/DEAD Fixable Violet Dead Cell Stain Kit (Thermo Fisher), and negative cells were considered viable. Cells were detected using LSR Fortessa (BD Bioscieces) and data analyzed with using 9.3.2 FlowJo software (Tree Star, Ashland, Oregon, USA).

### Patients and tissue samples

Twenty-four cases of metastatic melanoma were obtained from the Pathology Unit of the IRCCS Regina Elena National Cancer Institute. Three-micrometer sections of formalin-fixed paraffin-embedded tumor samples were cut on SuperFrost Plus slides (Menzel-Gläser, Braunschweig, Germany). Immunoreactions were revealed by Bond Polymer Refine Detection in an automated autostainer (Bond III, Leica Biosystems) using anti-bcl-2 (clone bcl-2/100/D5, Leica Biosystems) or -CD163 (Abcam) antibodies. Bcl-2 staining was classified in: score 0 (negative staining), score 1+ (staining that is faint/barely detectable), score 2+ (staining that is weak/moderate), score 3+ (staining that is intense/strong). Tumor-infiltrating M2 macrophages identified by CD163 were defined as cells with oval to round nuclei that showed strong membranous/cytoplasmic staining but not nuclear staining. The presence of CD163 positive cells was classiﬁed as PT and IT in four categories as described for F4/80 and CD206 staining in mice tumors. Evaluation of the IHC results was performed independently and in blinded manner by two investigators at 400× magnifications.

### Statistics

In vitro experiments were replicated at least three times, unless otherwise indicated, and the data were expressed as average±SD or ±SE of the mean (SEM). Differences between groups were analyzed with a two-sided paired or unpaired t test and were considered to be statistically significant for p<0.05. For in vivo experiments, the Mann-Whitney test was used to compare the mean score between the tumors in PT and IT area, while the Wilcoxon test was used to compare changes between the tumors in PT and IT evaluated 15 or 30 days after cell injection. The in vivo experiments were repeated twice by using at least five animals/group.

For IHC analysis on biopsies of patients with melanoma, the expression of bcl-2 was considered low when tumor cells exhibited a weak immunoreaction of the neoplastic cells (score 0, 1+), otherwise were defined as high (score 2+, 3+). We constructed a single variable for CD163 by adding the score values of the PT and IT. We thus obtained a distribution of values from 0 to 6, calculated the median value and created the definitive dichotomous variable based on this cut-off. We defined CD163 low if values were under the median values, otherwise were defined as high. Associations between variables were tested by Fisher exact test. P<0.05 were considered statistically significant. All statistical analyses were carried out with SPSS V. 21.0.

## Results

### Melanoma cells expressing bcl-2 promote migration and polarization of macrophages to a M2-type phenotype

**Figure 1:**
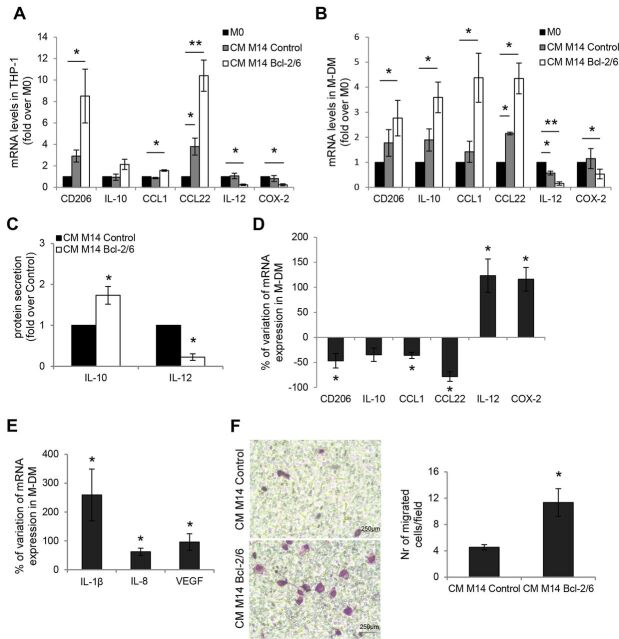
Melanoma cells expressing bcl-2 promote migration and polarization of macrophages to a M2-type phenotype. qRT-PCR analysis of CD206, IL-10, CCL1, CCL22, IL-12, COX-2 mRNA levels in (A) THP-1 cells and (B) human M-DM after 24 hours exposure to serum free medium (M0 macrophages) or to CM from M14 human melanoma control or bcl-2 overexpressing cells. (C) ELISA of IL-10 and IL-12 protein secretion in M-DM stimulated as reported in (B). (D) qRT-PCR analysis of CD206, IL-10, CCL1, CCL22, IL-12, COX-2 mRNA levels in M-DM stimulated with CM derived from M14 melanoma control or bcl-2 silenced cells. (E) qRT-PCR analysis of IL-1β, IL-8 and VEGF mRNA levels in M-DM stimulated as reported in (B). (F) Representative images (left panels) and relative quantification (right panel) of THP-1 cell migration in response to CM from M14 control (CM M14 Control) or bcl-2 overexpressing (CM M14 Bcl-2/6) melanoma cells. The values are reported as number of migrated cells/field. The quantification was performed by counting the number of migrated cells in at least 10 fields for each condition. The results are reported as fold induction relative to (A, B) M0 macrophages or to (C) melanoma control cells. The results are reported as % of mRNA variation in macrophages exposed to CM derived from (D) bcl-2 silenced cells versus control ones and from (E) bcl-2 overexpressing cells versus control ones. The average±SEM (A, B, D, E) or ±SD (C, F) of three independent experiments is reported. *P<0.05. **P<0.01. CM, culture medium; M-DM, monocyte-derived macrophages.

To test the effect exerted by melanoma-specific bcl-2 overexpression on macrophage functions, THP-1 monocytes were differentiated to macrophages (M0) and exposed for 24 hours to CM derived from parental melanoma M14 or A375SM-SC1 cells or their bcl-2 overexpressing counterparts (online supplementary figure S1A, B). CM from M14 parental cells partially modify the M1 and M2 markers in THP-1 (figure 1A) and M-DM (figure 1B) when compared with the baseline mRNA levels of M0 macrophages. More importantly, CM from both M14 (figure 1A) and A375SM-SC1 (online supplementary figure S1C) bcl-2 overexpressing cells induced a significant increase of mRNA expression of specific markers of M2 macrophages (CCL1, CCL22, IL-10) when compared with M0 macrophages, as opposed to a significant decrease of specific markers of M1 polarization, such as COX-2 and the antitumor cytokine IL-12. M2-polarizing activity of CM from bcl-2 transfectants derived from both M14 (figure 1B) and A375SM-SC1 (online supplementary figure S1D) cells was also demonstrated on M-DM. Accordingly, M-DM stimulated with CM from bcl-2 overexpressing cells showed increased IL-10 and decreased IL-12 protein production (figure 1C). We also evaluated the effect of CM derived from melanoma cells after silencing of bcl-2 with siRNA (si-bcl-2) (online supplementary figure S1E) on M-DM polarization. Noteworthy, the CM derived from both M14 (figure 1D) and A375SM-SC1 (online supplementary figure S1F) bcl-2 silenced cells significantly decreased mRNA levels of M2 markers and concomitantly increased M1 markers, when compared with M-DM stimulated with CM from si-control cells. Of note, M-DM exposed to CM from M14 bcl-2 overexpressing melanoma cells also increased the expression of IL-1β, IL-8, and VEGF, proteins known to improve tumor growth and vascularization^23^ (figure 1E). Interestingly, we also observed a significant increase of THP-1 cell migration in response to CM derived from bcl-2 overexpressing cells compared with CM from M14 (figure 1F) or A375SM-SC1 (online supplementary figure S1G) control cells.

10.1136/jitc-2019-000489.supp4 Supplementary data

### Bcl-2 overexpression in melanoma cells promotes diversion of macrophage functions toward a tumor-promoting phenotype

**Figure 2:**
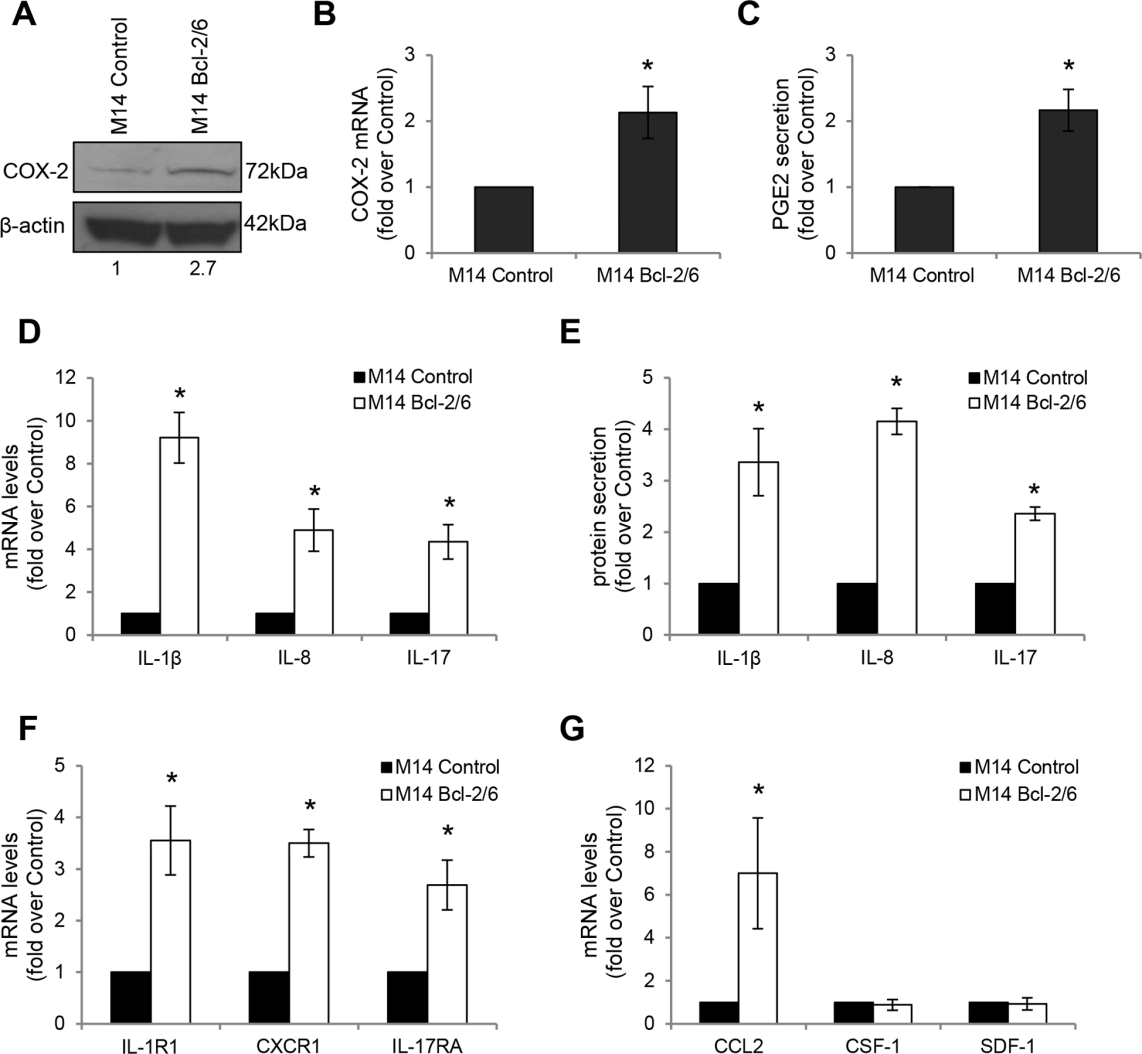
Bcl-2 drives expression of COX-2/PGE2 axis and IL-1β, IL-8, IL-17, CCL2 in melanoma cells. Analysis of COX-2 expression by (A) Western blot and (B) qRT-PCR analyses in M14 human melanoma control (M14 Control) and bcl-2 overexpressing (M14 Bcl-2/6) cells. (A) β-actin is shown as loading and transferring control. One representative western blot analysis out of two with similar results is reported. The numbers indicate densitometric analysis relative to control. (C) ELISA of PGE2 levels in CM derived from M14 control and bcl-2 overexpressing cells. PGE2 levels were normalized to the number of adherent cells. (D) qRT-PCR and (E) ELISA analyses of IL-1β, IL-8 and IL-17 expression in M14 control and bcl-2 overexpressing cells. (E) Protein levels were normalized to the number of adherent cells. (F) qRT-PCR analysis of IL-1β (IL-1R1), IL-8 (CXCR1), IL-17 (IL-17RA) receptors in M14 control and bcl-2 overexpressing cells. (G) qRT-PCR analysis of CCL2, CSF-1 and SDF-1 mRNA levels in M14 control and bcl-2 overexpressing cells. (B–G) Fold induction relative to control is reported. The results represent the average±SEM (B, D, F, G) or ±SD (C, E) of three independent experiments. *P<0.05.

Since PGE2 induces M2 macrophage polarization,^24^ and COX-2-dependent PGE2 production impairs recruitment and activation of immune cells,^25^ we investigated the effects of bcl-2 on the expression of COX-2/PGE2 by melanoma cells. Forced expression of bcl-2 in M14 (figure 2A–C) and A375SM-SC1 (online supplementary figure S2A, S2B) melanoma cells resulted in a significant increase of both protein and mRNA levels of COX-2, as well as secretion of PGE2. Interestingly, bcl-2 overexpressing cells displayed a significant upregulation of both mRNA and protein secretion of the inflammatory molecules IL-1β, IL-17 and IL-8, when compared with M14 (figure 2D, E) and A375SM-SC1 (online supplementary figure S2C) control cells. An increased expression of IL-1β (activatory IL-1R1), IL-17 (IL17RA) and IL-8 (CXCR1) receptors in bcl-2 overexpressing M14 cells was also observed (figure 2F).

10.1136/jitc-2019-000489.supp5 Supplementary data

Interestingly, an increased expression of CCL2, involved in macrophage recruitment,^26^ was observed in bcl-2 overexpressing M14 (figure 2G) and A375SM-SC1 (online supplementary figure S2D) cells, while no modulation of additional macrophage-recruiting factors, such as CSF-1 and SDF-1, was observed (figure 2G, online supplementary figure S2D).

To generalize the role of bcl-2 on TAM functions, we evaluated the effects of CM derived from H1299 lung cancer cells. As compared with H1299 control cells and similar to melanoma cells, CM from H1299 cells overexpressing bcl-2 promoted M2 polarization of M-DM, associated with increased expression of M2 (CD206, IL-10 and CCL1) and reduction of M1 (COX-2 and IL-12) markers (online supplementary figure S3A) and increased THP-1 cell migration (online supplementary figure S3B). Moreover, as demonstrated for melanoma cells, IL-1β, COX-2 and CCL2 were upregulated in bcl-2 overexpressing H1299 cells, while IL-8 was not affected and IL-17 was undetectable (online supplementary figure S3C). These findings indicate that bcl-2 levels in different tumor histotypes modulate macrophage migration and polarization and chemokines secretion.

10.1136/jitc-2019-000489.supp6 Supplementary data

**Figure 3:**
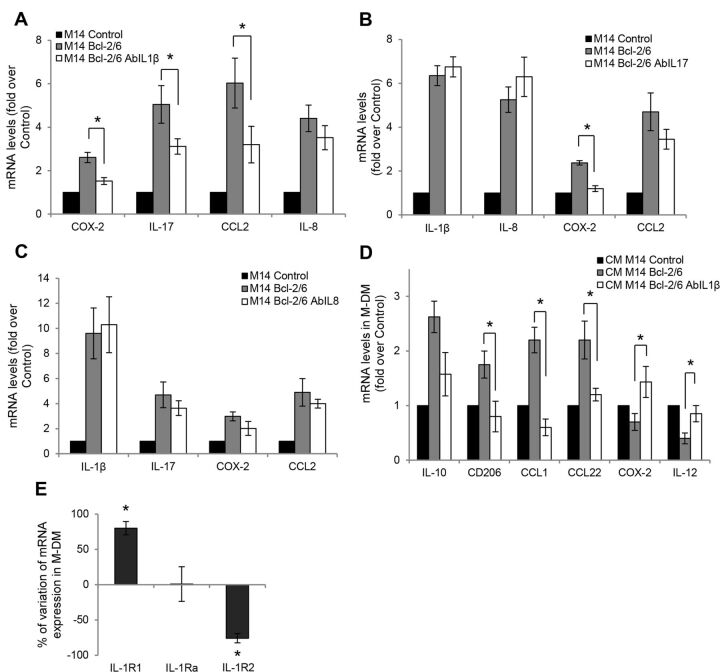
IL-1β secretion plays an autocrine role in controlling the expression of bcl-2-induced factors. qRT-PCR analysis of the indicated molecules in human melanoma M14 control and bcl-2 overexpressing cells untreated (M14 Bcl-2/6) or treated for 24 hours with (A) anti-IL-1β, (B) anti-IL-17, and (C) anti-IL-8 blocking antibodies (M14 Bcl-2/6 AbIL). (A–C) P values were calculated between bcl-2 overexpressing cells untreated and treated with antibody. Fold induction relative to control is reported. *P<0.05. (D) qRT-PCR analysis of the indicated molecules in M-DM stimulated with CM derived from M14 control, bcl-2 overexpressing cells untreated or treated for 24 hours with anti-IL-1β blocking antibody. P values were calculated between macrophages exposed to CM derived from bcl-2 overexpressing clone treated with anti-IL-1β blocking antibody versus bcl-2 overexpressing clone untreated. *P<0.05. (E) Analysis of mRNA levels of IL-1β (IL-1R1, IL-1Ra and IL-1R2) receptors in M-DM stimulated with CM from M14 control or bcl-2 overexpressing cells. The results are reported as % of mRNA variation in macrophages exposed to CM derived from bcl-2 overexpressing cells vs control one. *P<0.05. (A–E) The results represent the average±SEM of three independent experiments.

We next focused our attention to the melanoma models exposing bcl-2 overexpressing melanoma cells to either anti-IL-1β, anti-IL-17 or anti-IL-8 blocking antibodies and analyzing their impact on the expression of the identified bcl-2-inducible factors. Interestingly, IL-1β neutralization caused a significant reduction of COX-2, IL-17 and CCL2 expression, without affecting IL-8 mRNA levels (figure 3A). On the other side, IL-17 blocking antibody significantly reduced only the expression of COX-2 (figure 3B), whereas IL-8 neutralization failed to alter the expression of either IL-1β, IL-17, COX-2 or CCL2 (figure 3C). These results suggest that IL-1β secretion by melanoma cells plays an autocrine role in controlling the expression of bcl-2-induced COX-2, IL-17 and CCL2 levels.

To further determine possible paracrine effects of IL-1β on macrophages, we tested the impact of IL-1β blockade on the bcl-2-induced M2 polarization. CM derived from bcl-2 overexpressing melanoma cells treated with IL-1β blocking antibody caused a strong inhibition of M2 macrophage polarization, resulting in significant reduction of M2 (CD206, CCL1, CCL22) and induction of M1 (COX-2 and IL-12) markers (figure 3D). We also assessed the expression levels of IL-1β receptor subunits (IL-1R1, IL-1Ra, IL-1R2)^27^ in M-DM, following exposure to CM from melanoma cells. As reported in figure 3E, in M-DM stimulated with CM from bcl-2 overexpressing cells the expression of the activatory IL-1R1 subunit was significantly increased, whereas the expression of the decoy IL-1R2 strongly decreased. In contrast, expression of the IL-1β antagonist (IL-1Ra) was not affected. Thus, melanoma-released factors enhance the responsiveness of macrophages toward IL-1β by altering the ratio of expression of activatory *vs* inhibitory receptor subunits. Of note, an increased expression of IL-17 (IL-17RA), IL-8 (CXCR1) and CCL2 (CCR2) receptors was also observed on M-DM exposed to CM from bcl-2 overexpressing clone (online supplementary figure S2E), suggesting a larger impact of cancer cell-specific bcl-2 on the interplay between the tumor and the stromal compartment.

### Bcl-2 drives transcriptional activation of the IL-1β, IL-17, RORa, RORc and CCL2 genes in a NF-κB-dependent manner

**Figure 4:**
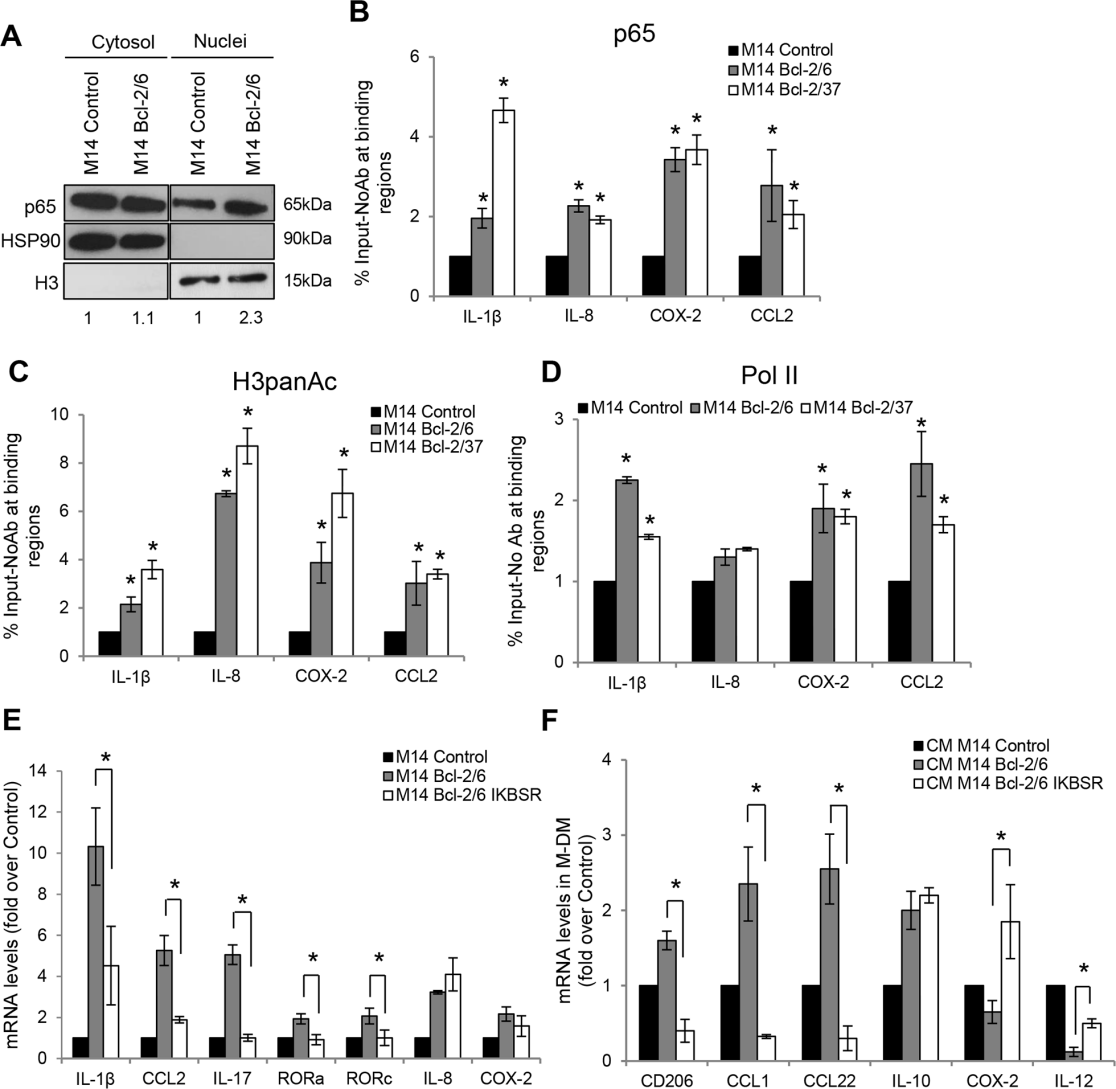
Bcl-2 drives transcriptional activation of IL-1β, IL-17, RORa, RORc and CCL2 in a NF-κB-dependent manner. (A) Western blot analysis of NF-κB subunit p65 in nuclear and cytosol extracts in M14 human melanoma control (M14 Control) and bcl-2 overexpressing clone (M14 Bcl-2/6). HSP90 and histone H3 are shown as loading, transferring and cytoplasmic/nuclear purification control. One representative western blot analysis out of two with similar results is reported. The numbers indicate densitometric analysis relative to control. ChIP analysis of (B) NF-κB subunit p65 recruitment, (C) histone H3 acetylation and (D) Pol II recruitment at IL-1β, IL-8, COX-2 and CCL2 promoters in M14 control and two bcl-2 overexpressing clones (M14 Bcl-2/6, M14 Bcl-2/37). The results are reported as % Input – NoAb. (E) qRT-PCR analysis of IL-1β, IL-8, IL-17, RORa, RORc, COX-2, CCL2 expression in M14 control, Bcl-2/6 and Bcl-2/6 IKBSR cells. (F) qRT-PCR analysis of CD206, IL-10, CCL1, CCL22, IL-12 and COX-2 expression in M-DM after exposure to CM from M14 control, Bcl-2/6 or Bcl-2/6 IKBSR cells. (B–F) Fold induction relative to control cells and the average±SEM of three experiments is reported. P values were calculated between (B–D) control and bcl-2 overexpressing cells or between (E, F) Bcl-2/6 cells and Bcl-2/6 overexpressing IKBSR cells, *P<0.05.

To unravel the molecular mechanism through which bcl-2 regulates the expression of factors related to macrophage polarization and migration, we focused our attention on NF-κB, a transcription factor implicated in the transcription of several bcl-2-induced molecules, including IL-1β,^28^ IL-8,^29^ IL-17,^30^ COX-2^31^ and CCL2.^32^ NF-κB regulation by bcl-2 was previously described by our group in breast cancer.^33^ Despite only a minor increase of p65 nuclear translocation was found in bcl-2 overexpressing M14 melanoma cells (figure 4A), ChIP analysis revealed an increased recruitment of p65 NF-κB member at proximal sites of IL-1β, IL-8, COX-2 and CCL2 promoters in bcl-2 transfectants (figure 4B). As a confirmation of these results, we further evaluated the acetylation level of histone H3 and the RNA Polymerase II (Pol II) enrichment at the IL-1β, IL-8, COX-2 and CCL2 promoters: bcl-2 overexpressing clones showed a significant increase of histone H3 acetylation at the four promoters (figure 4C), while Pol II enrichment was evident at the promoters of all the genes investigated, except for IL-8 (figure 4D).

Since NF-κB does not directly bind to IL-17 promoter, but regulates its expression through the transcription of RORa and RORc, two upstream transcriptional regulators of IL-17,^30^ the expression levels of RORa and RORc were tested. As reported in online supplementary figure S4A, mRNA levels of both RORa and RORc were significantly upregulated in bcl-2 overexpressing cells compared with control ones.

10.1136/jitc-2019-000489.supp7 Supplementary data

To assess the involvement of NF-κB in the bcl-2-mediated M2 macrophage polarization, we impaired the nuclear translocation of p65 by transfecting bcl-2 overexpressing melanoma cells with IKBSR, the mutated form of IKBα acting as NF-κB super repressor (online supplementary figure S4B, S4C). The impairment of NF-κB pathway in bcl-2 overexpressing M14 (figure 4E) and A375SM-SC1 (online supplementary figure S4D) cells, significantly reduced the IL-1β, IL-17, RORa, RORc and CCL2 mRNA levels, without significantly affecting IL-8 and COX-2 expression. Next, we explored the effect of impaired NF-κB activity on macrophage polarization: M-DM stimulated with CM from bcl-2 overexpressing M14 cells expressing NF-κB super repressor displayed a significant reduction of the M2 markers and induction of M1 ones, when compared with M-DM stimulated with bcl-2 overexpressing control transfected cells (figure 4F). Similar results were obtained with A375SM-SC1 bcl-2 overexpressing cells (online supplementary figure S4E). These findings indicate that the impairment of NF-κB pathway in bcl-2 overexpressing melanoma cells interferes with the bcl-2 ability to induce IL-1β, CCL2, IL-17 and its upstream regulators RORa and RORc, thus resulting in the failure of bcl-2-mediated macrophage reprogramming.

### Bcl-2 overexpressing melanoma tumors positively affect macrophage recruitment to the tumor site

To confirm the in vitro evidences, M14 control and bcl-2 overexpressing cells were injected in immunocompromised mice, observing a significant increase of tumor volume in bcl-2 overexpressing tumor bearing mice, starting from 22 days after cell injection (online supplementary figure S5A). Fifteen or 30 days after cells injection, the presence of IT or PT macrophages was analyzed by IHC analysis using the F4/80 antibody, a highly specific murine macrophage-related marker. Of note, 15 days after cells injection, a significant increased number of macrophages at the PT areas was observed in bcl-2 overexpressing tumors (mean score: 1.4±0.5 for control vs 2.6±0.5 for bcl-2 overexpressing tumors, p=0.032) (online supplementary figure S5B, S5D). When IHC analysis was performed 30 days after cell injection, the bcl-2 overexpressing xenografts showed a significant increase in the number of both IT (mean score: 0±0 for control vs 2.8±0.4 for bcl-2 overexpressing tumors, p=0.008) and PT (mean score: 1.8±0.8 for control vs 3±0 for bcl-2 overexpressing tumors, p=0.032) macrophages (online supplementary figure S5B, S5D). To characterize the status of in vivo macrophage polarization, we also performed IHC analysis for the specific M2 marker CD206. A significantly increased expression of CD206 at IT level (mean score: 0±0 for control vs 2.3±0.5 for bcl-2 overexpressing tumors, p=0.007) was observed in bcl-2 overexpressing tumor xenografts when compared with control ones (online supplementary figure S5C, S5E). As expected and reported in online supplementary figure S5C, higher levels of bcl-2 protein were observed in tumor xenografts obtained after injection of bcl-2 overexpressing cells when compared with control tumors, thus confirming that bcl-2 expression was maintained during in vivo tumor growth.

10.1136/jitc-2019-000489.supp8 Supplementary data

**Figure 5:**
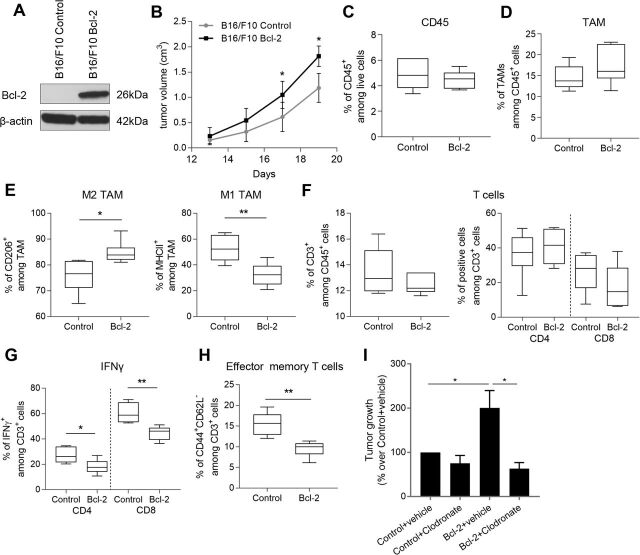
Melanoma-specific bcl-2 establishes a suppressive microenvironmental condition that impairs T cell response. (A) Western blot analysis of bcl-2 protein expression in murine melanoma B16/F10 control (B16/F10 Control) or bcl-2 overexpressing (B16/F10 Bcl-2) cells. β-actin is shown as loading and transferring control. One representative western blot analysis out of two with similar results is reported. (B) Quantification of in vivo tumor growth after subcutaneous injection of B16/F10 control or bcl-2 overexpressing cells in C57/Bl6 mice. Quantification by cytofluorimetric analysis of (C) CD45^+^ cells among live cells, (D) cd11b^+^F4/80^+^ (TAM) among CD45^+^ cells, (E) CD206^+^ (left panel), and MHCII^+^ (right panel) cells among TAM in B16/F10 control or bcl-2 overexpressing tumors. Quantification by cytofluorimetric analysis of (F) CD3^+^ among CD45^+^ cells (left panel), CD4^+^ and CD8^+^ among CD3^+^ cells (right panel), (G) IFNγ production and (H) CD44^+^CD62L^-^ among CD3^+^ infiltrating cells. (G) The expression level of IFNγ was analyzed after stimulation with brefeldin A, ionomycin, and PMA as reported in Methods section. (I) Quantification of in vivo tumor growth of C57/Bl6 mice subcutaneously injected with control (Control) or bcl-2 overexpressing (Bcl-2) B16/F10 cells and treated with vehicle or with clodronate liposomes. Tumor growth is expressed as % over Control+vehicle group. (C–H) The results were reported as % of positive cells and represent the average ±SD of two independent experiments. (B–H) P values were calculated between control and bcl-2 overexpressing tumors. *P<0.05; **p<0.01. (I) P values were calculated between control and bcl-2 overexpressing tumors treated with vehicle or between bcl-2 overexpressing tumors treated with vehicle or clodronate. *P<0.05.

To validate our results in an immunocompetent context and to assess the effect of bcl-2 expression on T cells, we injected B16/F10 murine melanoma control and bcl-2 overexpressing cells (figure 5A) in C57/Bl6 mice. We observed a significant increase of tumor volume in bcl-2 overexpressing tumor bearing mice, starting from 17 days after cell injection (figure 5B). Nineteen days after tumor injection, we performed immune phenotype analysis of tumors: even if flow cytometric analysis revealed a comparable level of infiltrating leukocytes (CD45^+^) (figure 5C, online supplementary figure S6A) and TAM (cd11b^+^F4/80^+^, figure 5D, online supplementary figure S6B) between control and bcl-2 overexpressing allografts, a significant increase of the M2 marker CD206 (figure 5E, left panel, online supplementary figure S6C), along with a decrease of the M1 marker MHCII (figure 5E, right panel, online supplementary figure S6D), was observed in bcl-2 overexpressing tumors. In contrast, no differences were observed on the number of tumor infiltrating T cells (CD3^+^) (figure 5F, left panel, online supplementary figure S6E), as well as CD4^+^ and CD8^+^ T cell subsets (figure 5F, right panel, online supplementary figure S6F).

10.1136/jitc-2019-000489.supp9 Supplementary data

**Figure 6:**
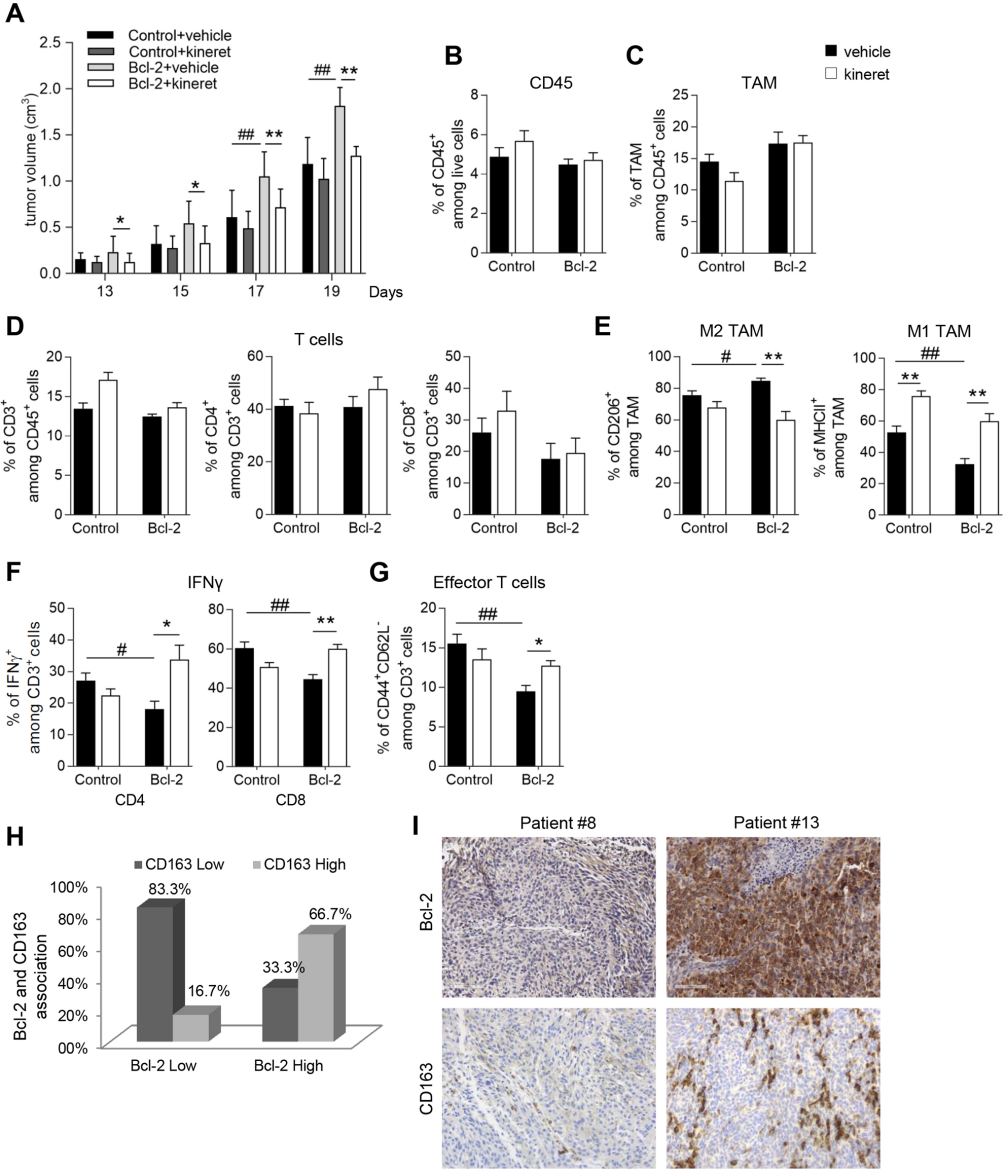
IL-1β plays a central role in the bcl-2-mediated effect on TAM and T cell functions. (A) Quantification of tumor volume in C57/Bl6 mice subcutaneously injected with control (Control) or bcl-2 overexpressing (Bcl-2) B16/F10 cells and treated with vehicle or with kineret (1 mg/kg, daily) from day 3 to day 12. Quantification by cytofluorimetric analysis of (B) CD45^+^ cells among live cells, (C) cd11b^+^F4/80^+^ (TAM) among CD45^+^ cells, (D) CD3^+^ among CD45^+^ cells (left panel), CD4^+^ (middle panel) and CD8^+^ (right panel) among CD3^+^ cells, (E) CD206^+^ (left panel), and MHCII^+^ cells (right panel) among TAM in B16/F10 control or bcl-2 overexpressing tumors treated with vehicle or with kineret as reported in (A). Quantification by cytofluorimetric analysis of (F) IFNγ production and (G) CD44^+^CD62L^-^ among CD3^+^ infiltrating cells in B16/F10 control or bcl-2 overexpressing tumors treated with vehicle or with kineret as reported in (A). The results were reported as % of positive cells. (H) Quantification of bcl-2 and CD163 association in metastatic melanoma specimens, p=0.036. (I) Representative images of IHC analysis of bcl-2 and CD163 expression in patient #8 (bcl-2 low; CD163 low) and patient #13 (bcl-2 high; CD163 high). (A–G) The results represent the average ±SD of two independent experiments. P values were calculated between control and bcl-2 overexpressing tumors (#) and between tumors treated with vehicle or kineret (*), *p<0.05; **p<0.01; #p<0.05; ##p<0.01.

Interestingly, both CD4^+^ and CD8^+^ cells from bcl-2 overexpressing tumor produced lower levels of interferon γ (IFNγ) compared with control ones (figure 5G, online supplementary figure S6G). Accordingly, a significant decrease in the frequency of effector memory T cells (CD44^+^CD62L^-^) was observed in bcl-2 overexpressing tumors (figure 5H, online supplementary figure S6H). Collectively, our in vivo results corroborate the in vitro data on the bcl-2 ability to induce the recruitment of M2 macrophages and indicate the ability of cancer-specific bcl-2 to establish suppressive microenvironmental conditions that impair T cell responses.

To explore the role of TAMs in the bcl-2 mediated effect in vivo, we treated C57/Bl6 mice carrying parental or bcl-2 overexpressing B16/F10 tumors with clodronate liposomes, in order to deplete macrophages. Interestingly, at 18 days after cell injection, clodronate treatment significantly reduced the growth of bcl-2 overexpressing tumors to a level similar to those of control tumors, while any significant reduction was observed in the growth of parental tumors (figure 5I). Nevertheless, clodronate was also able to delay the tumor appearance in mice. In particular, while at 8 days after bcl-2 overexpressing cell injection, the 100% of mice treated with vehicle developed tumors, the percentage of tumors was only 62.5% after clodronate treatment. This percentage increased to 75%, 75% and 87% when tumor appearance was evaluated at days 11, 14 and 18, respectively (table 1). The IHC analysis of F4/80^+^ cells confirmed the macrophage depletion in bcl-2 overexpressing tumor bearing mice (online supplementary figure S6I).

To validate in vivo the central role of IL-1β in the bcl-2-mediated effect on TAM and T cell functions, we investigated the effect exerted by IL-1β signaling blockade, by treating mice with kineret, a recombinant form of human IL-1 receptor antagonist (IL-1Ra). Surprisingly, kineret treatment significantly reduced the growth of bcl-2 overexpressing tumors, while only a marginal and not significant inhibition was observed in control tumors (figure 6A). Moreover, while kineret failed to modulate the percentage of infiltrating leukocytes (CD45^+^) (figure 6B), TAM (cd11b^+^F4/80^+^) (figure 6C) and lymphocytes (CD3^+^, CD4^+^ or CD8^+^) (figure 6D) either in control or in bcl-2 overexpressing tumors, it drastically affected the phenotype of both TAM and T cells in bcl-2 overexpressing tumors. In particular, kineret reverted the M2 polarization of TAM infiltrating bcl-2 overexpressing tumors, as evident by the decreased expression of M2 marker CD206 and the concomitant increase of M1 marker MHCII (figure 6E). Moreover, a significant increase of IFNγ production by CD4 (figure 6F, right panel) and CD8 T (figure 6F, left panel) cells, along with an increased number of effector memory T cells (figure 6G), was observed in bcl-2 overexpressing tumors treated with kineret.

To assess the relevance of our observation in patients with melanoma, we performed a retrospective analysis by using a collection of 24 metastatic melanoma biopsies, associating the bcl-2 expression with the M2 marker CD163. In agreement with the in vitro and in vivo preclinical results, we observed a strong correlation between bcl-2 and CD163. In particular, low levels of bcl-2 were associated with low CD163 expression (figure 6H, I left columns) while, conversely, high bcl-2 levels correlated with high expression of CD163 (figure 6H, I right columns) (p=0.036).

## Discussion

In this manuscript, we evaluated whether the genetic background of tumor cells dictates the nature and modality of interactions with components of the tumor microenvironment, such as macrophages. We demonstrate that cancer-specific bcl-2 promotes an IL-1β-driven pathway supporting the recruitment of M2 polarized macrophages, as well as formation of a pro-tumor microenvironment. In particular, melanoma cells expressing high levels of bcl-2 displayed enhanced activation of the IL-1β/COX-2 axis, which paralleled an increased gene expression and secretion of IL-8, IL-17 and CCL2. In turn, these phenotypic changes affected macrophage functions by enhancing their expression of tumor-promoting factors (ie, IL‐1β, IL-8 and VEGF), known to regulate malignant progression, through increased angiogenesis, leukocyte recruitment and tumor cell invasion.^12 34 35^ The relevance of this observation is sustained by previous works showing that the COX-2/PGE2 axis is a major player in cancer development and growth, acting through induction of angiogenesis and tumor invasiveness,^36^ promotion of M2 macrophage polarization^37^ and impaired recruitment and activation of immune cells.^38^

We also confirmed the role of bcl-2 on TAM functions in non-small cell lung cancer histotype.

Within the bcl-2-driven protumor reprogramming, IL-1β acts as critical upstream regulator of tumor growth and macrophage functions, since its neutralization decreased the expression of COX-2 and IL-17, as well as expression of M2 polarization markers by macrophages. In agreement, an IL-1β/COX-2/PGE2 positive feedback was reported in breast cancer cells and macrophages.^39^

IL-1β has been reported to exert its protumorigenic functions through interaction with IL-1R1 on stromal and tumor cells, through both paracrine and autocrine signaling.^40^ IL-1β is produced by macrophages in order to support tumor growth and immune tolerance^41 42^ and by stromal cells to promote metastasis through M2 type macrophages^43^ and blocking its pathway led to growth arrest in IL‐1β‐positive melanoma cells^44^ and reversion of immunosuppression in breast cancer.^45^ Moreover, IL-1 was suggested as possible mediator for the activation of the IL-1R/MyD88/IKKβ pathway, reported to induce M2 polarization of bone marrow-derived macrophages.^24^

As both melanoma cells and macrophages exposed to supernatants from bcl-2-overexpressing cells expressed higher levels of the receptors for IL-1β, IL-17, IL-8 and CCL2, it is conceivable that these cytokines may critically support an active interplay between the tumoral and stromal compartments.

In a first attempt to elucidate the molecular mechanism orchestrated by bcl-2, we focused our attention on NF-κB, a key transcription factor directly or indirectly involved in the transcription of several molecules induced by bcl-2 overexpression in our models, including IL-1β,^28^ IL-8,^29^ IL-17,^30^ COX2^31^ and CCL2.^32^ We demonstrated the involvement of NF-κB in both the expression of bcl-2-induced factors and bcl-2-mediated M2 macrophage polarization. The evidence that in cancer models bcl-2 strongly supports the recruitment of NF-κB,^33^ MITF,^7^ Sp1,^46^ Hypoxia Inducible Factor 1α,^5^ c-Myb^47^ and SUFU/GLI^48^ at their DNA binding sites on the promoter of MMP9, TRPM1, uPAR, VEGF, Semaphorin 5A and anti-apoptotic genes respectively, indicates a general phenomenon of bcl-2 regulation in the activity of different transcription factors. Even if striking inconsistencies have been reported for the expression of bcl-2 with melanoma progression,^49 50^ our results reflect an increased malignant potential by bcl-2 overexpressing melanoma cells and are in agreement with those studies evidencing a protumoral role of bcl-2 and a positive correlation between bcl-2 expression and melanoma progression.^50^

In support of bcl-2 as cancer-associated orchestrator of TAM recruitment and functions, a massive in vivo recruitment of M2-polarized macrophages was observed both in human and murine melanoma models injected in mice. In the syngeneic mouse model, we evidenced the ability of cancer-specific bcl-2 to impair T cell response, through reduced production of IFNγ and the effector memory T cells population. This evidence indicates a link between bcl-2-driven diversion of macrophage functions and impaired specific antitumor immunity. In further agreement, treatment of melanoma-bearing mice with kineret, a recombinant human IL-1 receptor antagonist, confirmed IL-1β as a critical factor controlling bcl-2 regulation of immune cells, since it significantly reduced tumor growth and expression of the M2 marker CD206 by TAM, while in contrast, it enhanced the IFNγ production by both CD4 and CD8 T cells as well as the number of tumor-infiltrating effector memory T cells. Finally, in tumor specimens from patients with melanoma, high bcl-2 expression correlated with increased infiltration of M2-polarized CD163^+^ TAM, hence supporting the clinical relevance of tumor/microenvironment crosstalk.

## Conclusion

Our work provides the first demonstration of a complex crosstalk between cancer-specific bcl-2 and TAM, through the involvement of NF-κB and identifies IL-1β as central player of the bcl-2-driven protumor reprogramming of macrophages, as well as orchestrator of the functional crosstalk between cancer and immune cells. Overall, our study suggests the view that inhibition of IL-1β, along with NF-κB activity, may lead to a therapeutic M2 to M1 switch of macrophage polarization and reactivation of specific antitumor immunity, hence supporting the use of COX inhibitors (eg, aspirin) for the prevention and treatment of cancer immunosuppression.^38 51^ Even if results of clinical trials with bcl-2 inhibitors in melanoma were not encouraging in the past and inadequate patient selection was recognized as a limiting factor to reach better results,^2^ inhibition of bcl-2 or bcl-2-driven IL-1β should potentially be considered in combination with immune checkpoint inhibitors for the treatment of selected melanoma, especially those expressing high level of bcl-2 or not involving BRAF mutations.
