# Supplementary material for: Melanoma-specific bcl-2 promotes a protumoral M2-like phenotype by tumor-associated macrophages
Source: J Immunother Cancer. 2020 Apr 7;8(1):e000489. doi: 10.1136/jitc-2019-000489 (PMC7254128; doi:10.1136/jitc-2019-000489)
Supplement: Supplementary data [file jitc-2019-000489supp001.pdf]

ONLINE SUPPLEMENTARY FILES LEGENDS

Online supplementary Table S1. Primers used for qRT-PCR analysis.

Online supplementary Table S2. Primers used for ChIP analysis.

Online supplementary Figure S1. (A-B) Western Blot analysis of Bcl-2 protein expression in **(A)** M14 and (**B**) A375SM-SC1 control cells and their bcl-2 overexpressing derivatives. β-actin is shown as loading and transferring control. Representative Western Blot analyses out of two with similar results are reported. **(C,D)** qRT-PCR analysis of CD206, IL-10, CCL1, CCL22, IL-12, COX-2 mRNA expression in (**C**) THP-1 cells and in (**D**) human monocyte-derived macrophages (M-DM) after 24h exposure to CM derived from bcl-2 overexpressing or control A375SM-SC1 cells. (**E**) qRT-PCR analysis of bcl-2 mRNA levels in M14 and A375SM-SC1 melanoma cells transfected with siRNA control (si-control) or siRNA against bcl-2 (si-bcl-2). (**F**) qRT-PCR analysis of CD206, IL-10, CCL1, CCL22, IL-12, COX-2 mRNA expression in M-DM exposed to CM derived from A375SM-SC1 cells transfected as in (**E**). **(G)** Representative images (left panel) and relative quantification (right panel) of migrated THP-1 cells in response to culture medium (CM) derived from A375SM-SC1 control (CM A375SM-SC1 Control) or bcl-2 overexpressing (CM A375SM-SC1 Bcl-2) melanoma cells. The values are reported as number of migrated cells/field. The quantification was performed by counting the number of migrated cells in at least 10 fields for each condition. The average ± SD of three independent experiments is reported. (**C,D,F**) The results are reported as % of mRNA variation in macrophages exposed to CM derived from (**C,D**) bcl-2 overexpressing cells versus control one and from (**F**) bcl-2 silenced versus control cells. The average ± SEM of three independent experiments is reported. p-values were calculated between macrophages exposed to CM from (**C,D**) control and bcl-2 overexpressing cells, or from (**F**) control and bcl-2 silenced cells. *$p<0.05$.

Online supplementary Figure S2. COX-2, PGE2, IL-1β, IL-8, IL-17 and CCL2 expression in A375SM-SC1 control and bcl-2 overexpressing cells. **(A)** qRT-PCR analysis of COX-2 expression in A375SM-SC1 control and bcl-2 overexpressing cells (Bcl-2). **(B)** ELISA of PGE2

levels in cultured medium (CM) from A375SM-SC1 control and bcl-2 overexpressing cells. PGE_2 levels were normalized to the number of adherent cells. Results are reported as average ± SD of three independent experiments. **(C, D)** qRT-PCR analysis of (**C**) IL-1β, IL-8 and IL-17 and (**D**) CCL2, CSF-1 and SDF1 mRNA expression in A375SM-SC1 control and bcl-2 overexpressing cells. (**E**) Analysis of mRNA levels of IL-17 (IL-17RA), IL-8 (CXCR1) and CCL2 (CCR2) receptors in M-DM stimulated with cultured medium from M14 control or bcl-2 overexpressing cells. The results are reported as % of mRNA variation in macrophages exposed to CM derived from bcl-2 overexpressing cells versus control one. *p<0.05. **(A, C-E)** The results represent the average ± SEM of three independent experiments. **(A-D)** Fold induction of bcl-2 overexpressing cells relative to control cells is reported. *p<0.05.

Online supplementary Figure S3. Macrophages differentiation and migration after exposure to CM from H1299 parental and bcl-2 overexpressing cells. (**A**) mRNA levels of the indicated molecules by qRT-PCR in M-DM exposed for 24h to CM derived from H1299 human non-small cell lung carcinoma control (CM H1299 Control) or bcl-2 overexpressing (CM H1299 Bcl-2) cells. The results are reported as % of mRNA variation in macrophages exposed to CM derived from bcl-2 overexpressing cells versus control ones. (**B**) Representative images (left panel) and relative quantification (right panel) of THP-1 cell migration in response to CM derived from H1299 control or bcl-2 overexpressing cells. The values are reported as number of migrated cells/field. The quantification was performed by counting the number of migrated cells in at least 10 fields for each condition. (**C**) qRT-PCR analysis of IL-1β, IL-8, COX-2 and CCL2 expression in H1299 control and bcl-2 overexpressing cells. Fold induction relative to control is reported. The results represent the average ± SEM (**A,C**) or ± SD (**B**) of three independent experiments. *p<0.05.

Online supplementary Figure S4. Involvement of NF-κB in IL-1β, IL-8, IL-17, COX-2 and CCL2 expression in A375SM-SC1 cells and in macrophage differentiation. (**A**) qRT-PCR analysis of RORa and RORc mRNA levels in M14 control and bcl-2 overexpressing cells (M14 Bcl-2/6). Western Blot analysis of IKBα protein level in (**B**) M14 and (**C**) A375SM-SC1control

cells, bcl-2 overexpressing bcl-2 overexpressing cells (Bcl-2) and bcl-2 overexpressing cells transiently transfected with IKBSR (Bcl-2 IKBSR). HSP72/73 or HSP90 are shown as loading and transferring control. One representative Western Blot analysis out of two with similar results is reported. (**D**) qRT-PCR analysis of IL-1β, IL-8, IL-17, COX-2 and CCL2 expression in A375SM-SC1 control cells, bcl-2 overexpressing cells (Bcl-2) and bcl-2 overexpressing cells transiently transfected with IKBSR (Bcl-2 IKBSR). (**E**) qRT-PCR analysis of CD206, IL-10, CCL1, CCL22, IL-1β, IL-12 and COX-2 expression in human monocyte-derived macrophages after exposure to cultured medium from A375SM-SC1 control, bcl-2 overexpressing cells (Bcl-2) and bcl-2 overexpressing cells transiently transfected with IKBSR (Bcl-2 IKBSR). (**A,D,E)** Fold induction relative to control cells is reported and the average ± SEM of three experiments is reported. p-values were calculated between (**A**) control and bcl-2 overexpressing cells or between (**D,E**) Bcl-2/6 cells and Bcl-2/6 overexpressing IKBSR cells, $*p<0.05$.

Online supplementary Figure S5. Macrophage recruitment and polarization in control and bcl-2 overexpressing tumors. (A) *In vivo* tumor growth after subcutaneous injection of M14 control or bcl-2 overexpressing cells in nude mice. Representative images (**B,C**) and relative quantification (**D,E**) of peritumoral (PT) and intratumoral (IT) infiltration of (**B**) F4/80 and (**C**) CD206 and bcl-2 staining by IHC analysis of M14 control and bcl-2 overexpressing (M14 Bcl-2/6) tumors performed 15 or 30 days after cell injection. The results are reported as mean score. Score 0: no detectable infiltrate; score 1: low infiltrate; score 2: moderate infiltrate; score 3: high or very high infiltrate. Each dot (•) indicates an experimental point. 5 animals for each condition were evaluated. (**A-E**) The experiments have been repeated two times. p-values were calculated between control and bcl-2 overexpressing tumors, $*p<0.05$; $**p<0.01$.

Online supplementary Figure S6. Immune infiltrate recruitment in control and bcl-2 overexpressing tumors.

Representative Flow Cytometry graphs of (**A**) $CD45^+$ cells among live cells, (**B)** $cd11b^+F4/80^+$ (TAM) among $CD45^+$ cells, (**C**) $CD206^+$ and (**D**) $MHCII^+$ cells among TAM, (**E**) $CD3^+$ among

$CD45^+$ cells, (**F**) $CD4^+$ and $CD8^+$ among $CD3^+$ cells, (**G**) IFNγ production and (**H**) $CD44^+CD62L^-$ among $CD3^+$ infiltrating cells in B16/F10 control or bcl-2 overexpressing tumors. (**I**) IHC analysis of F4/80 in B16/F10 control and bcl-2 overexpressing (Bcl-2) tumors treated with vehicle or with clodronate. Analysis was performed 18 days after cell injection.
