# Supplementary material for: Melanoma-specific bcl-2 promotes a protumoral M2-like phenotype by tumor-associated macrophages
Source: J Immunother Cancer. 2020 Apr 7;8(1):e000489. doi: 10.1136/jitc-2019-000489 (PMC7254128; doi:10.1136/jitc-2019-000489)
Supplement: Supplementary data [file jitc-2019-000489supp002.pdf]

GENE	FORWARD (5'-3')	REVERSE (5'-3')
**BCL-2**	CTGCACCTGACGCCCT5TCACC	CACATGACCCCACCGAACTCAAAGA
**IL-10**	GCGCTGTCATCGATTTCTTC	TGGCTTTGTAGATGCCTTTCTC
**IL-12**	GAGGCCTGTTTACCATTGGA	TCAAGGGAGGATTTTTGTGG
**CD206**	GCTGTTCTCCTACTGGACACCA	AATCTGAGATTCGGACACCCA
**COX-2**	GTTCCACCCGCAGTACAGAA	AGGGCTTCAGCATAAAGCGT
**IL-1β**	TGGCCCTAAACAGATGAAGTGC	CTGAAGCCCTTGCTGTAGTGGT
**CCL1**	AAGAGCATGCAGGTACCCTTCT	CTCATTGGAGCAGATGGAGCT
**CCL22**	TGGCGCTTCAAGCAACTGA	AAGTGTTTCACCACGCGCA
**IL-1R1**	ACAAGGCCTTCTCCAAGAAGAATA	TGCATTTATCAGCCTCCAGAGA
**IL-17**	TGGAATCTCCACCGCAATGA	GCTGGATGGGGACAGAGTTC
**IL-8**	CACCGGAAGGAACCATCTCA	TGGCAAAACTGCACCTTCACA
**IL-17RA**	CGAATGGACACTGCAGACAGAC	CAAACCTGACGCACAAACGTT
**CXCR1**	TCAACCCCATCATCTACGCCT	TGACGTGCCAAGAACTCCTTG
**IL-1R2**	TTCCGCTTGTACGTGTTGGT	TTGTAATGCCTCCCACGAAAC
**IL-1Ra**	CCAGCTGGAGGCAGTTAACATC	GCAGACTCAAAACTGGTGGTGG
**CCL2**	TCTCGCCTCCAGCATGAAAGT	GCATTGATTGCATCTGGCTGA
**SDF-1**	ACACTCCAAACTGTGCCCTT	CTGTAAGGGTTCCTCAGGCG
**CSF-1**	ATGCGCTTCAGAGATAACACCC	ATAGAAAGTTCGGACGCAGGC
**VEGF**	TCTTCAAGCCATCCTTGTTG	TCTGCATGGTGATGTTGGAC
**CCR2**	GGAGAGCAGAGAGTGGAAATGT	GTGAAGGCGGAGATACAGGG
**RORa**	TTTCCCTACTGTTCGTTCACCA	GTTTGGCAAACTCCACCACA
**RORc**	GCCTCAGCTTTGACCTGTCTCA	ATGTCTTGGTCCCCCAGAAGTC
**β-actin**	ATTGCCGACAGGATGCAGAA	GCTGATCCACATCTGCTGGAA
