# Supplementary material for: Melanoma-specific bcl-2 promotes a protumoral M2-like phenotype by tumor-associated macrophages
Source: J Immunother Cancer. 2020 Apr 7;8(1):e000489. doi: 10.1136/jitc-2019-000489 (PMC7254128; doi:10.1136/jitc-2019-000489)
Supplement: Supplementary data [file jitc-2019-000489supp003.pdf]

**IL-1β**	CTGTGTGTCTTCCACTTTGTCC	AATCGTTGTGCAGTTGATGTCC
**IL-8**	GAAAACTTTCGTCATACTCCG	GAAAGTTTGTGCCTTATGGAG
**COX-2**	AGACATCTGGCGGAAACCTG	AACCAAGCCCATGTGACGAA
**CCL2**	CAGCAGATTTAACAGCC	TTCTGGGTTAGTCTCAGC
