# Supplementary figures and images for: Melanoma-specific bcl-2 promotes a protumoral M2-like phenotype by tumor-associated macrophages
Source: J Immunother Cancer. 2020 Apr 7;8(1):e000489. doi: 10.1136/jitc-2019-000489 (PMC7254128; doi:10.1136/jitc-2019-000489)

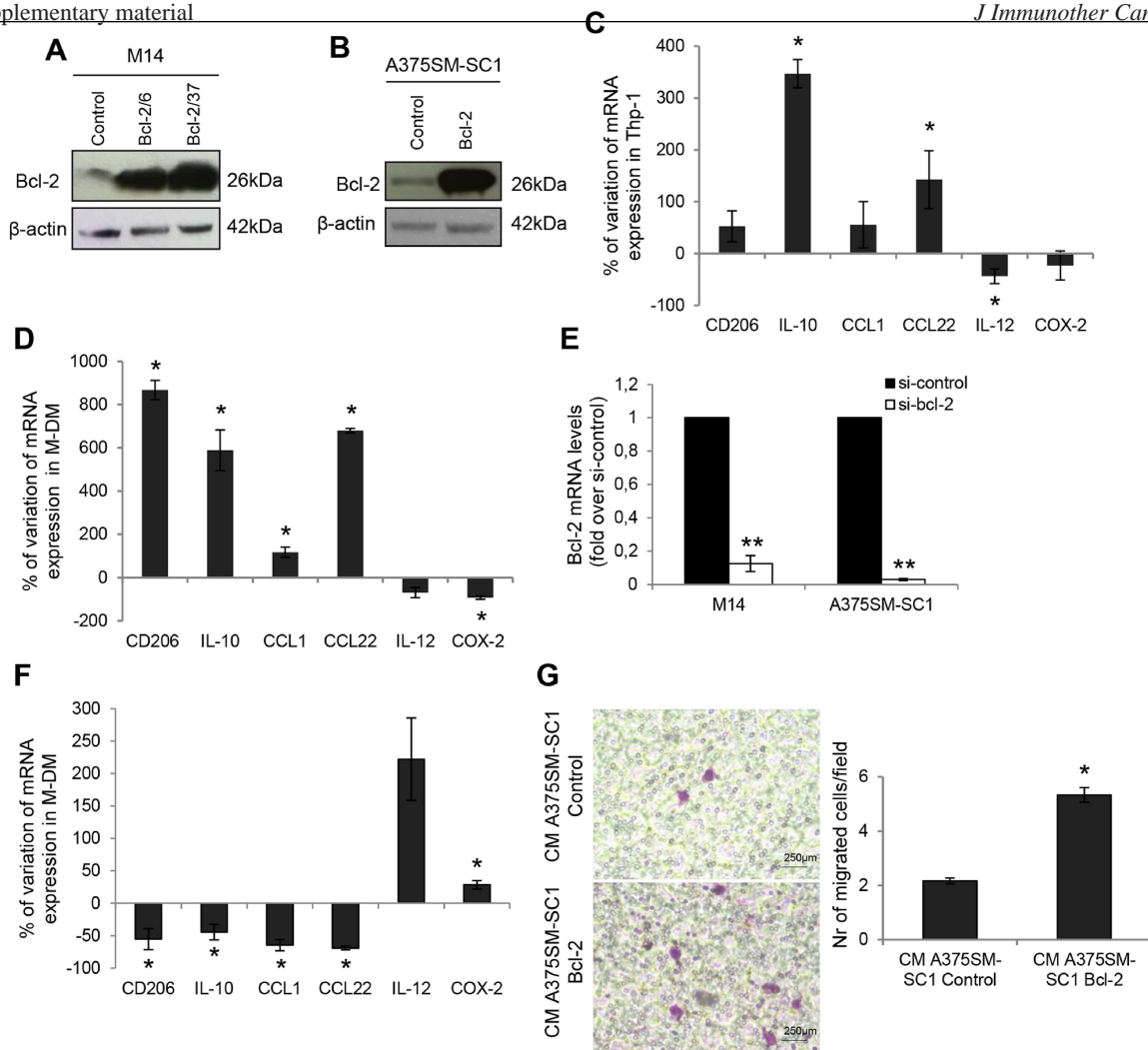

Supplement: Supplementary data [file jitc-2019-000489supp004.pdf]

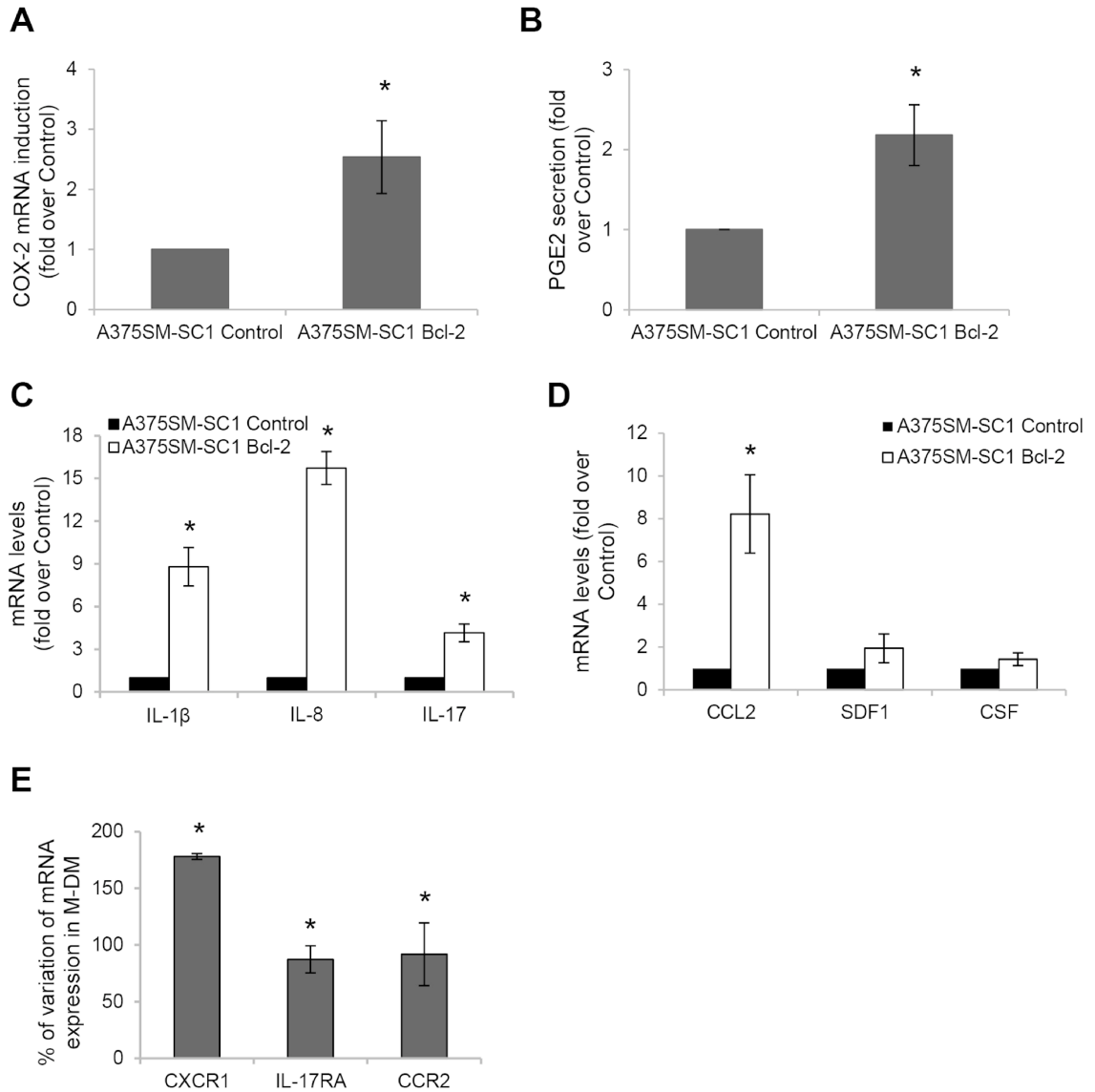

Supplement: Supplementary data [file jitc-2019-000489supp005.pdf]

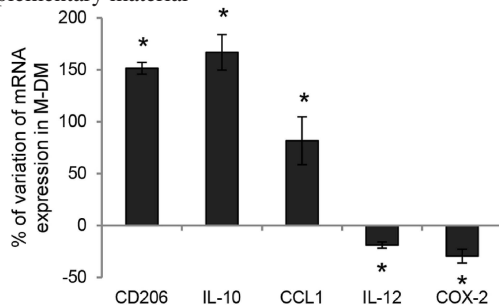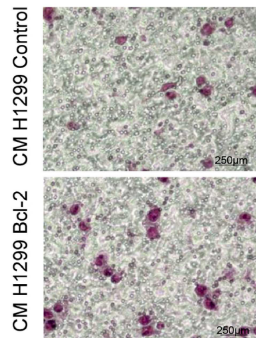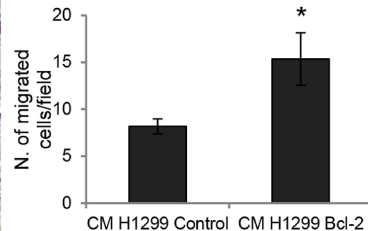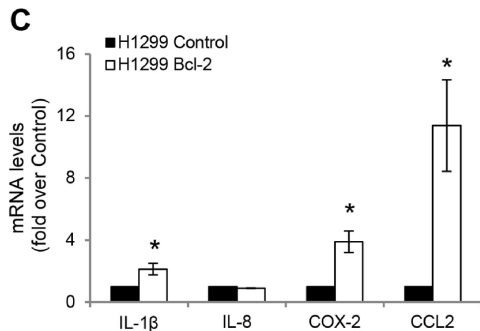

Supplement: Supplementary data [file jitc-2019-000489supp006.pdf]

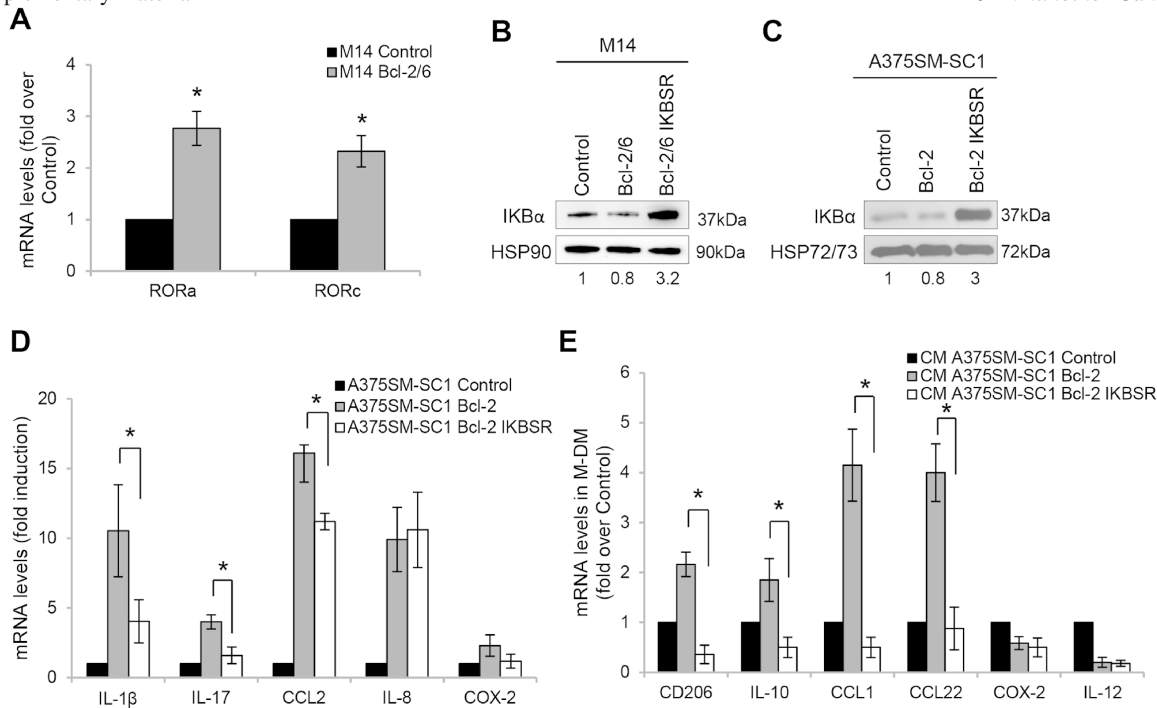

Supplement: Supplementary data [file jitc-2019-000489supp007.pdf]

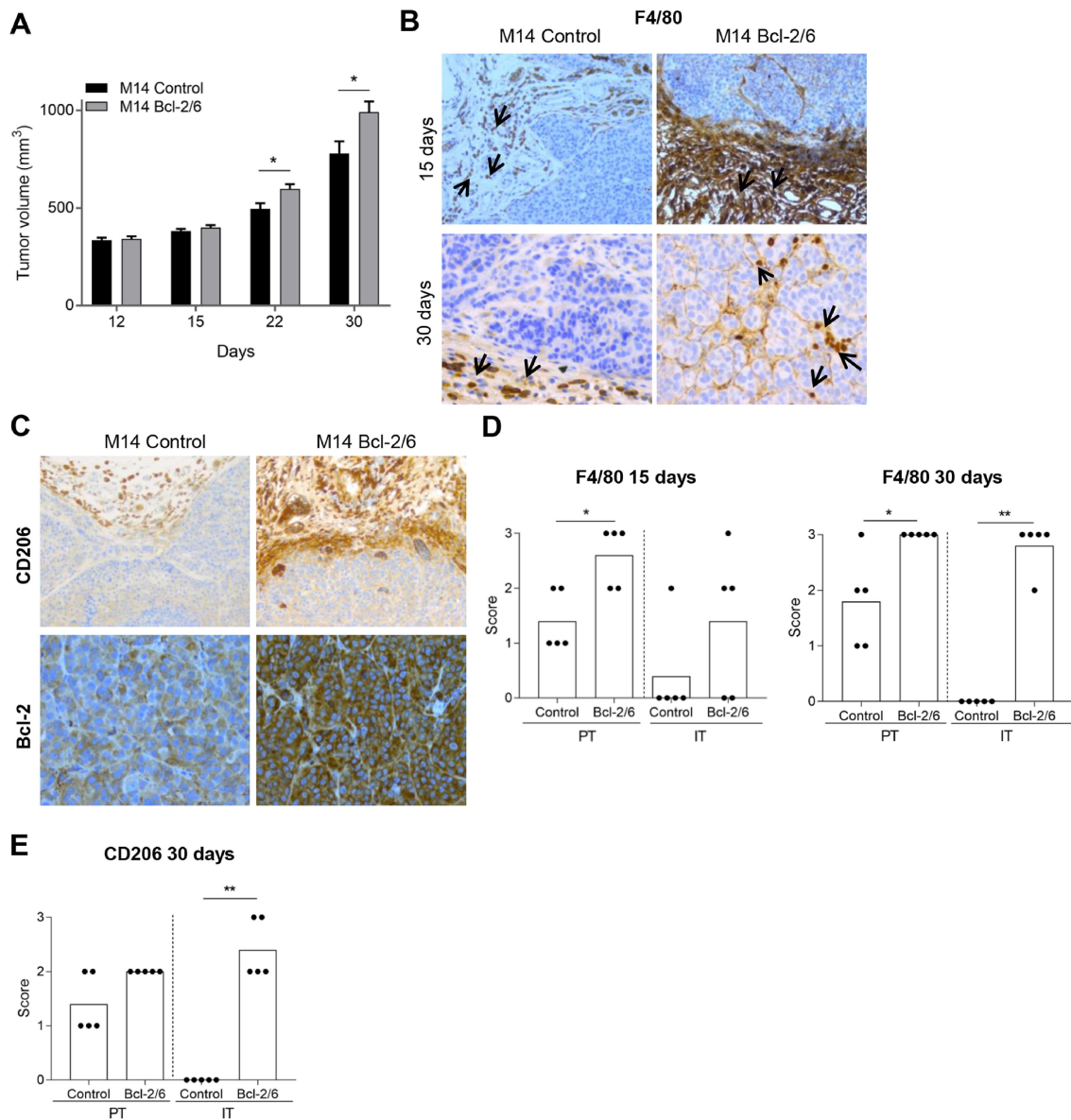

Supplement: Supplementary data [file jitc-2019-000489supp008.pdf]

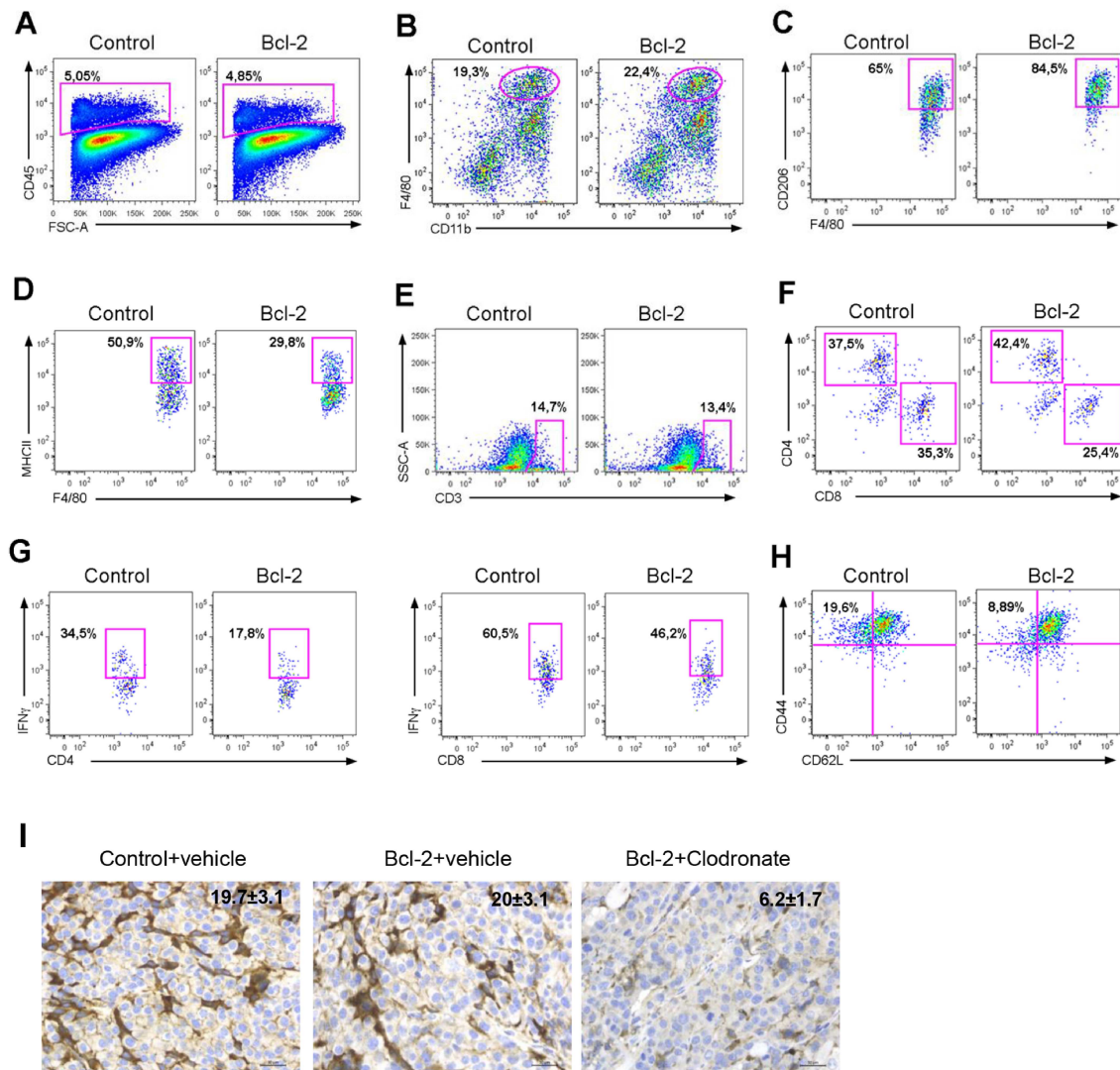

Supplement: Supplementary data [file jitc-2019-000489supp009.pdf]
